# Mozambique field epidemiology and laboratory training program: a pathway for strengthening human resources in applied epidemiology

**DOI:** 10.11604/pamj.2017.27.233.13183

**Published:** 2017-07-31

**Authors:** Cynthia Semá Baltazar, Cátia Taibo, Jahit Sacarlal, Lorna Gujral, Cristolde Salomão, Timothy Doyle

**Affiliations:** 1 National Institute of Health, Maputo, Mozambique; 2 Faculty of Medicine, Eduardo Mondlane University; 3 National Public Health Directorate, Ministry of Health, Mozambique; 4 Centers for Disease Control and Prevention, Mozambique

**Keywords:** Mozambique, field, epidemiology

## Abstract

**Introduction:**

In the last decades, Mozambique has been undergoing demographic, epidemiological, economic and social transitions, which have all had a notable impact on the National Health System. New challenges have emerged, causing a need to expand the preparation and response to emerging disease threats and public health emergencies.

**Methods:**

We describe the structure and function of the Mozambique Field Epidemiology Training Program (MZ-FELTP) and the main outputs achieved during the first 6 years of program implementation (consisting of 3 cohorts). We also outline the contribution of the program to the National Health System and assess the retention of the graduates.

**Results:**

The MZ-FELTP is a post-graduate in-service training program, based on the acquisition of skills, within two tracks: applied epidemiology and laboratory management. The program was established in 2010, with the objective of strengthening capacity in applied epidemiology and laboratory management, so that events of public health importance can be detected and investigated in a timely and effective manner. The program is in its seventh year, having successfully trained 36 health professionals in the advanced course. During the first six years of the program, more than 40 outbreaks were investigated, 37 surveillance system evaluations were conducted and 39 descriptive data analyses were performed. Surveillance activities were implemented for mass events and emergency situations. In addition, more than 100 oral and poster presentations were given by trainees at national and international conferences.

**Conclusion:**

The MZ-FELTP has helped provide the Ministry of Health with the human and technical resources and operational capacity, to rapidly and effectively respond to major public health challenges in the country. The continuous involvement of key stakeholders is necessary for the continuation, expansion and ongoing sustainability of the program.

## Introduction

The epidemiological profile of Mozambique is characterized by infectious diseases that are closely related to poverty and other socio-economic factors prevailing in the country [1]. The major causes of morbidity and mortality are malaria, HIV/AIDS, tuberculosis, respiratory diseases, diarrheal disease, measles, and meningitis [1, 2]. The country is vulnerable to natural disasters (e.g cyclones, floods) and frequent outbreaks of infectious diseases. Outbreaks of cholera and other epidemic diseases recur every year [3, 4]. Currently, the country is facing a demographic transition, with non-communicable diseases increasing in frequency, adding pressure on the health system [2]. The epidemiological surveillance system was first established by the Mozambican Ministry of Health in 1979 and underwent revisions in 1985 and 2005 [1]. There were two assessments of the surveillance system, one in 2001 and the other in 2006. Among the identified constraints of the surveillance system was the lack of human resource capacity of health personnel for disease surveillance, reporting, data management, public health preparedness and response [1, 5]. Timely response to infectious disease outbreaks still challenges the National Health System [1]. In addition, there is an insufficient number of qualified public health professionals and they are not equitably distributed throughout the country [1, 2, 5].

Furthermore, few health professionals in the country are using epidemiological skills, such as routine analysis and presentation of data. The Mozambican Ministry of Health thus identified the need for training of multidisciplinary technical teams where epidemiologists and laboratory technicians are members of surveillance and outbreak investigation teams. In this context, the Mozambique Field Epidemiology and Laboratory Training Program (MZ-FELTP) was created in 2009 by the National Institute of Health (INS), in partnership with the National Directorate of Public Health (DNSP) and the Faculty of Medicine of Eduardo Mondlane University (UEM), with technical and financial support from the U.S Centers for Disease Control and Prevention (CDC). Similar to other Field Epidemiology Training Programs, the MZ-FELTP is modeled on the Epidemic Intelligence Service (EIS) of the CDC. The EIS was modeled on a post-graduate residency program. It combined classroom and applied training and mentored in-service activities to train professionals in applied epidemiology and disease control, through the motto of "learn by doing". A global network of programs was established in 1997 as the Global Training Programs in Epidemiology and Public Health Interventions Network (TEPHINET) [6, 7]. The African Field Epidemiology Network (AFENET) was established in 2004, after recognizing the opportunities on the African continent for improving epidemiologic capacity through a network of country and region-specific training programs [8–10]. The MZ-FELTP is a member of the AFENET and TEPHINET professional networks. Connections and collaborations within these professional networks have gradually strengthened since the initiation of MZ-FELTP. These networks are a professional and scientific platform for the exchange of knowledge and experience.

## Methods

This manuscript describes the program and includes a review of the main outputs. The outputs include outbreak and field investigations, surveillance system evaluations, descriptive data analyses, documentation of standard laboratory procedures, conference presentations, training activities and publications. The proportion of graduates retained in the National Health System was also assessed.

## Results

**FELTP training model in Mozambique**: The aim of MZ-FELTP is to build epidemiological capacity in public health surveillance, disease control and response to outbreaks and public health emergencies through training in applied intervention epidemiology and laboratory management. To do this, the MZ-FELTP provides both a short-term, basic course and an advanced master's level course, affiliated with UEM. The master's course is 2 years duration, with approximately 25% of the time spent in classroom modules at the UEM Department of Microbiology and 75% spent in field placement activities. The classroom portion is organized into four modules (Introduction to Public Health and Basic Epidemiology, Scientific Communication and Research Processes, Advanced Epidemiology and Laboratory Management in Public Health and Management and Leadership in Public Health) with each module lasting approximately 3-6 weeks. The remaining 75% of time, residents are placed in field sites where they develop their skills and competencies in core activities of learning through supervised day-to-day activities at their field site. Field sites are strategic locations such as provincial health departments, national programs (malaria, HIV, non-communicable diseases, neglected tropical diseases and others), central cross-cutting departments at the Ministry of Health (epidemiology department and others) and reference laboratories at the National Institute of Health (tuberculosis, serology, microbiology, virology and molecular biology) and other key areas related to health (e.g Department of Animal Sciences).

During the master's program, each resident is assigned a mentor who guides the resident during the two years of training. Mentors help review the resident's work outputs and provide technical and professional guidance. At each field location, a field supervisor provides ongoing day-to-day oversight on the experiential learning activities. A two-day training is offered for the mentors and supervisors every year, where their roles and responsibilities are described. The FELTP coordinating group performs regular field site visits and meets with both mentors and supervisors to ensure ongoing coordination and support of the training process. Residents enrolled in the master's program are placed in one of two tracks, based on their background, experience and future professional goals. These are the epidemiology track or laboratory management track. During the two years, residents in both tracks acquire expertise in epidemiological methods, biostatistics, public health surveillance, public health laboratory practice, scientific communication, management and leadership and emergency response.

Classroom modules make extensive use of case studies and practical exercises. Additional training methodologies include topic specific workshops, seminars and journal clubs. Residents are evaluated through written tests and evaluation of their program outputs. In addition to the academic activities of the four core modules, the curriculum is flexible to include additional training opportunities on specific issues. There have been several training activities on specific topics including workshops on non-communicable disease, risk communication, molecular epidemiology of infectious diseases, scientific writing, HIV/AIDS and geographic information systems (GIS), among others. The MZ-FELTP is governed by an Executive Steering Committee that is led by the National Public Health Director and includes senior-level officials from the Ministry of Health (MoH). The steering committee also includes representatives from key partner organizations such as CDC, WHO and UEM. This steering committee meets once or twice a year and has the role of advising and guiding the program on issues related to the program objectives, training quality, finances, trainee recruitment and program advocacy.

### Timeline of key developments of the MZ-FELTP

**Short courses in basic epidemiology**: The aim of the basic epidemiology short course is to strengthen basic public health surveillance at the district level, providing staff with the epidemiological tools to solve problems in the community. The first short course was initiated in 2009, targeting district-level health professionals who regularly participate in epidemiological surveillance activities, outbreak control and laboratory activities. Several additional courses were conducted in subsequent years (Table 1). In general, selection of the participants in the course is the responsibility of each Provincial Health Directorate (DPS). The course generally has 20-25 participants and a duration of two weeks, except for the first two short courses that were three months duration with a field work component and mentorship. In the first four short courses, the participants came from several provinces and the course was held at the central level (Maputo City). However, the approach was changed during the fifth course, where the training was held in each province and with only participants of that province (Table 1). In addition to reducing the costs of the course, this also allowed course content to be more focused on the health priorities of each Province. The longer-term training plan for the epidemiologic short courses in Mozambique envisions one to two courses per year in different regions of the country, so that each province is reached every three to four years.

**Table 1:** Implementation of 8 basic epidemiology short courses, Mozambique, 2009-2015

Year	Number of participants	Target Provinces*
2009	24	Maputo Province, Maputo City, Gaza, Cabo Delgado
2010	18	Maputo, Gaza, Sofala, Nampula, Zambézia and Niassa
2012	26	Maputo Province, Maputo City and Gaza
2012	24	Maputo Province, Gaza
2013	20	Cabo Delgado
2014	18	Niassa
2015	25	Zambézia
2015	25	Nampula
Total	180	

* 8 of 11 provinces covered

**Advance course-masters level**: The 2-year advanced, master's level FELTP course was implemented in August 2010, with the first residents enrolled in the program. The first group was composed of 11 residents, of which five enrolled in the epidemiology track and six in the laboratory track (Table 2). Several residents in the first cohort participated in the 2009 epidemiology short course and in this way, the different levels of training are designed to be synergistic with one another. In March 2012, the second class cohort began with 14 residents, of which nine were enrolled in the epidemiology track and five in the laboratory track. The third cohort started in March 2014, with 12 residents (eight in epidemiology track and four in the laboratory track). Enrollment in the program is based on a competitive process where MoH first announces the call for applications in major newspapers. Then selection involves review of application materials and evaluation of applicant skills by a selection committee made up of program staff and key stakeholder organizations. The master's degree is provided by UEM Faculty of Medicine.

**Table 2:** Current profile of MZ-FELTP graduates

				Current Position (in 2017)				
Cohort	Track	Admission	Completed	MoH	Provincial Health Department	UEM	MoD	NGO
**Cohort 1** (2010-12)	Epi	5	4 (80%)	2	2	0	0	0
	Lab	6	6 (100%)	5	0	1	0	0
**Cohort 2** (2012-14)	Epi	9	9 (100%)	7	2	0	0	0
	Lab	5	5 (100%)	2	1	1	1	1
**Cohort 3** (2014-16)	Epi	8	8 (100%)	2	4	0	1	1
	Lab	4	4 (100%)	1	3	0	0	0
Total Percentage		37	36 (97%)	19 (51%)	12 (32%)	2 (5.5%)	2 (5.5%)	1 (2.7%)

UEM: University Eduardo Mondlane MoD: Ministry of Defense NGO: Non-government organization

**Program accomplishments**: Although the MZ-FELTP is a relatively young program, it has contributed to notable strengthening of human resources in the health system in Mozambique. The program has been able to respond to epidemiological necessities such as conducting more than 40 outbreak investigations and 37 surveillance system evaluations. Other important field activities conducted by program participants include: independent monitoring of a mass polio vaccination campaign; evaluation of coverage and impact of a malaria bed-net distribution campaign; response to natural disasters such as catastrophic flooding and surveillance during mass gatherings (Table 3). The MZ-FELTP program has also enhanced Mozambique's contribution to the dissemination of epidemiological information through the publication of 11 articles published in the MoH Epidemiological Bulletin related to outbreak investigations and descriptive analyses of surveillance data (Table 3). There have also been approximately 100 oral and poster presentations at national and international scientific conferences; four manuscripts were published in international journals [11–14], with additional manuscripts in preparation or in press. The MZ-FELTP residents played a key role in important field activities. This includes supporting public health surveillance for mass gatherings during the All-African Games held in Maputo in September 2011, through the implementation of a syndromic surveillance system. Residents coordinated the collection, compilation and epidemiological analysis of data from the major health service centers during the event to detect and respond in a timely manner to any outbreaks that might occur.

**Table 3:** Major outputs of MZ-FELTP, 2010-2016

Outputs	Quantity	Topics
Outbreak investigations and response	42	Measles, cholera, rotavirus, typhoid, polio, poisoning, shigellosis, influenza, conjunctivitis, malaria, dengue, chikungunya, and yellow fever
Field work (Non-outbreak investigation)	6	Evaluation of polio and measles vaccination campaigns, evaluation of mosquito nets distribution campaigns, response to emergencies and surveillance at mass events (African Games)
Descriptive analyses of surveillance data	39	Cancer, TB-MDR, antimicrobial resistance, water and food, vaccine coverage , measles , rubella, occupational health, leprosy, exposure to HIV, nutrition, neonatal tetanus, lymphatic filariasis, malaria, cholera, community health workers, non-communicable disease, and neglected tropical diseases
Articles in the Epidemiological Bulletin	14	Outbreak investigations and descriptive surveillance data analysis
Creation of database	8	Laboratory information system
Creation of laboratory standard operating procedures	27	Laboratory procedures
Surveillance system evaluation	37	AFP, measles, HIV anti-natal surveillance, TB, malaria, water and food, polio, lymphatic filariasis , non-communicable diseases , mortality, dysentery, cholera, diarrhoea, immunization coverage, leprosy, laboratory information system, trauma,
Oral presentations at scientific conferences	>70	International: AFENET, TEPHINET, EPISUS National: Mozambique National Scientific Conference on Health, Mozambique Ministry of Science and Technology Conference, Maputo Central Hospital Scientific Conference, UEM Scientific Conference on Health Scientific Conference, INS Open Days for Research
Poster presentations at scientific conferences	>30	International: AFENET, TEPHINET, EPISUS National: Health days, MCT, HCM
Training activities	>24	Workshops: Non-communicable diseases, risk communication, molecular epidemiology of infectious diseases, scientific writing
Journal publications	4	International peer-reviewed journal

In this case, no outbreaks or unusual disease patterns were identified; however, the activity provided valuable information and reassurance to event organizers and the public during a complex mass-gathering. In addition, catastrophic flooding affected Gaza Province in 2013, resulting in the displacement of an estimated 120,000 inhabitants. During this natural disaster, MZ-FELTP residents conducted real-time surveillance for events of public health importance affecting the displaced population. Increases in diarrheal disease and malaria were noted among the displaced population and this real-time surveillance informed emergency response agencies and facilitated timely implementation of disease control and response measures. An important indicator of success of the program is the graduation and retention of graduates in the National Health System (Table 2). Of the 37 students who enrolled in the three cohorts, 36 (97%) successfully completed the program. All graduates were allocated to the national health system, through different programs at central and provincial levels, at the National Institute of Health reference laboratories and at the Ministry of Defense (Military Hospital Laboratory of Maputo). In addition, two residents were sponsored by UEM and they continue working there. An external assessment was conducted in 2013 using a scorecard that was developed by CDC and applied to FETP/FELTP evaluations in multiple other countries. The tool includes five domains relevant to successful FELTP implementation: training, field work, leadership development, management and sustainability. The assessment evaluated the implementation and performance of the program and identified key areas for program improvement. Further program evaluation at periodic intervals is anticipated, as is done with other FELTP programs globally and those accredited through TEPHINET.

## Discussion

Following establishment of the program, progress has been made in strengthening the response to outbreaks and improvements of disease surveillance systems and activities in the country. The majority of MZ-FELTP graduates have been retained in the Mozambique Health System. This is similar to the FELTPs in other African countries [10, 15–17]. Although there is strong graduate retention, the Ministry of Health needs to further develop a well-defined career path for the graduates in epidemiology and public health laboratory management, to optimize the impact and utility of this human resource cadre and ensure ongoing retention. The program's sustainability is an ongoing challenge, since it is an intensive training program requiring significant human and financial resources. Currently, FELTP in Mozambique is mostly funded by the CDC, with some contributions from the Ministry of Health. The long-term success of FELTPs depends on their sustainability [16], thus the government entities and institutions in host countries must assume ownership of the program and diversify funding with multisector support. One of the priorities is the strengthening of field placements to ensure that the development of skills and competencies are in-line with program objectives and academic training goals. The existence of mentors and qualified field supervisors is crucial for the success of this training model. The limited dissemination and communication of research results through publication in international and national journals suggests the need to create strategies for strengthening scientific writing. However, language also poses a challenge which has been addressed through support of technical advisers who are native English speakers who can review outputs.

## Conclusion

Epidemiology is an essential pillar of public health and as such, must be closely linked to policies, programs and services. While MZ-FELTP is still at a relatively early stage in its growth and development, it has shown some positive and significant impacts which are assisting the country to better detect and respond to outbreaks and address major public health challenges. It is an important platform for developing a multidisciplinary and cohesive workforce, with trained health professionals that can operate effectively in various segments of the public health system to improve health security. Continued engagement and involvement of all stakeholders and program partners is necessary to ensure sustainability and expansion of the program beyond its current scope.

### What is known about this topic

- The FELTP curriculum has been adopted in many African countries to strengthen the public health system;
- The FELTP model is unique for its emphasis of on-the job training methodology prioritizing “learning by doing”;
- The program has been documented for providing an effective response to public health emergencies, including disease outbreaks and other public health events.

### What this study adds

- This is the first article describing the experience of FELTP in Mozambique;
- This article reports the main successes and challenges of FELTP in Mozambique;
- This manuscript reinforces the importance of key stakeholder engagement for the continuation, expansion and ongoing sustainability of the program.

## Competing interests

The authors declare no competing interest.
